# Working towards a consensus for antibody validation

**DOI:** 10.12688/f1000research.5668.1

**Published:** 2014-11-05

**Authors:** Peter D. Reiss, Danxi Min, Mei Y. Leung

**Affiliations:** 1 St John’s Laboratory, London, SE8 5RT, UK; 2 PeproTech EC Ltd, London, W6 8LL, UK

## Abstract

Commercial research antibodies are the most commonly used product in the life science tools market, and their applications represent a significant investment of time and resources for researchers. Frequently however, the quality of antibodies does not meet the expectations of consumers, causing loss of valuable time and money. This can delay research efforts and scientific discovery, or even lead to false, irreproducible results to be published in the scientific literature. This raises the question of whether there should be universal standards for validating antibodies.

During the 1 ^st^ International Antibody Validation Forum, hosted by St John’s Laboratory Ltd on October 15 ^th^ 2014 at Queen Mary University of London, scientists from academia and industry presented data highlighting quality issues arising from lack of antibody validation. While the forum identified significant current problems in the antibody market, it also discussed future opportunities for improved quality and transparency by encouraging data disclosure and data sharing. This article highlights the key issues and conclusions reached at the forum.

## Addressing the need for standards

In his keynote talk entitled “Use and Abuse of Antibodies in the Lab and in The Clinic”, Dr David Rimm, Professor of Pathology at Yale University, showed alarming examples of non-reproducible studies due to un-validated antibodies ^1, 2^. According to Dr Rimm, the most commonly reported problem users of antibodies encounter is non-specificity, or cross-reactivity; the binding of an antibody to not only the epitope of its target protein, but also to epitopes of homologous proteins. This and other validation issues, including evaluating sensitivity and reproducibility were clearly presented as a framework for a rigorous approach to validating antibodies. In 2010, Dr Rimm introduced his antibody validation algorithm for immunohistochemistry (IHC) and immunofluorescence (IF) ^3^. The need for standardisation guidelines controlling antibody variability was alluded to in another paper showing variability of commercial antibodies to three cancer-related biomarkers ^4^. It was found that most of the antibodies against EGFR recognising different epitopes of full-length protein did not correlate well. To date, there are no standardisation guidelines to control for antibody variability in IHC biomarker measurement studies. It is recommended that these guidelines include validation for specificity, sensitivity and reproducibility and incorporate the following criteria: a) Sensitivity: Test signal to noise on FFPE (formalin-fixed paraffin embedded) test, TMAs (tissue microarrays) at multiple titers; b) Specificity *in vitro*: Western blot, IP or knock-down; c) Specificity on the slide: Linearity in FFPE cell line series or siRNA knock-down; d) Reproducibility: Regressions of multiple lot, multiple day testing on serial TMA sections.

Irreproducibility in preclinical research using antibodies can ultimately be traced to an absence of a unifying framework of standards and best practices. Consensus on standards for demonstrating validity of IHC proficiency in biomarker studies would greatly improve reproducibility, particularly in the area of cancer research, and accelerate the translation of research breakthroughs into life-saving therapies.

## Balancing cost and quality for suppliers

With the large numbers of commercially available antibodies, it is difficult for customers to navigate the market landscape and to compare different products. Chief Scientific Officer at Everest Biotech, Dr Jan Voskuil, ^5^ discussed the problems in the Other External Manufacturer (OEM) system, in which there is one manufacturer producing one product that becomes rebranded into various brands by many different suppliers. This results in the same product appearing several times on the same catalogue. The main problem with this system is that under the OEM agreement, the vendors can keep their quality control (QC) data that was generated with a different batch of antibodies. With significant batch-to-batch variations in specificity, especially among polyclonal antibodies, many of the QC data can be misleading for researchers. Ideally, each new batch should be subject to quality control as if it was a new antibody ^4^.

In agreement with Everest Biotech, Dr John Mountzouris, site leader at Abgent, pointed out that suppliers have a moral duty to provide proof of the antibody’s quality, which is needed by the consumer to make an informed choice. His view from the supplier’s side addressed the issue of integrity with focus on disclosure of product information. The expectation of the customers is not only for the antibody to work the first time, but also to have validated proof of its quality. However, in most cases, data from commercial antibodies do not include sufficient information and does not reflect the batch-to-batch differences. When Abgent performed a screening of their antibodies with western blot, several thousand antibodies had to be discontinued immediately. Although the company saw an initial revenue decline, customer complaint rates dropped below a low of 5 percent. Due to the high cost associated with antibody validation, companies’ business decisions will invariably impact their level and degree of validation.

## New antibody array technology for antibody testing

The potential for smaller suppliers to gain market share was discussed by Dr Fridtjof Lund-Johansen, who is a principal investigator at the Institute of Immunology at Oslo University, Norway. The current antibody market suffers from lack of head to head validation comparison, which leads to the concept of survival of the first rather than survival of the best. Dr Lund-Johansen has developed a highly sophisticated antibody array that can measure and compare antibody binding in large numbers ^6^. This proteomics based technology called microsphere affinity proteomics (MAP) allows peptide binding to antibodies conjugated to fluorescent beads and is a fraction of the cost of traditional methods. When used to compare high ranking antibodies with antibodies that had no and few citations, the results were surprisingly revealing. The less cited antibodies were often found to perform better than the blockbusters. Would companies now use this technology to test their catalogue antibodies?

## Independent Antibody Validation

The need for independent validation was also discussed by Dr Andrew Chalmers, lecturer at the University of Bath and founder of CiteAb, a citation based antibody search engine. Simply put, suppliers cannot possibly generate all information by themselves, especially since an antibody is typically used for different applications and tissues. Furthermore, it was suggested that journals should add antibody reporting guidelines, instructing authors to include antibody reports ^7^. The antibody search engine and review database pAbmAbs, ( http://pabmabs.com/wordpress/) presented by founder Dr Simon Glerup, also provides a useful platform for researchers to share antibody reports. These reviews have helped Dr Glerup and his team to confidently identify the right antibody for his experiments within neuroscience research.

## Peer-Reviewed Antibody Validation

As researchers often lack information about how a particular antibody performs under specific conditions and application, the forum welcomed the launch *of F1000Research’*s new online open access Antibody Validation Collection ( http://f1000research.com/article-collections/antibody-validation). Associate publisher of *F1000Research*, Michael Markie discussed how this online publishing platform offers a new transparent approach to post-publication peer review with full data deposition and sharing. The permanent article collection on Antibody Validation provides a platform to publish antibody validation studies and disseminate valuable antibody information to researchers based on actual use cases of the antibodies of interest. *F1000Research* has established a set of publication guidelines and appointed an editorial board from both academia and industry. The main message from each article is how a particular antibody performed in a particular setting, as well as specific information about the antibody itself, such as catalogue number, batch number, target peptide and full experimental details. In each publication, the complete results must be included revealing how the antibody performed in different repeats and conditions. Authors are expected to provide full details of methodology to allow reproducibility under the same research conditions. In addition, this collection accepts cases of failed validation and negative findings, data that traditionally has been difficult to publish. A peer-reviewed collection of antibody validation articles holds significant potential, and not only allows for reproducibility but would give the scientific community an incentive to validate antibodies to the level and standard acceptable to leading scientists.

## Are we close to reaching a consensus?

The forum concluded with promising opportunities for future improvements on how we can validate, select, and use antibodies with more confidence. However, real change and success will begin when both end users and suppliers start to adopt and apply these principles. Although biomedical products and therapies used in clinical research are highly regulated, the use of antibodies in basic and pre-clinical research have no compliance requirements for commercialisation. Standardisation and written consensus for antibody information disclosure and minimum acceptable level of antibody validation is likely to lessen the occurrence of erroneous findings. However, unlike the drug industry, the development of life science standards must first gain widespread acceptance. Life science standards and the dilemma of irreproducible experiments have recently been the focus of GBSI (Global Biological Standards Institute) an independent body for the advancement of medical science through the development and advocacy of biological and life science standards ^8^. Given the positive responses received from the participants, it is highly likely that a follow-up forum will take place. This will hopefully lead to the establishment of the forum as a regular event and facilitate the implementation of antibody validation standards.
